# Notes on the Particulate Matter Standards in the European Union and the Netherlands

**DOI:** 10.3390/ijerph6031155

**Published:** 2009-03-17

**Authors:** Hugo Priemus, Elizabeth Schutte-Postma

**Affiliations:** 1 OTB Research Institute for Housing, Urban and Mobility Studies / Delft University of Technology / PO Box 5030, 2600 GA Delft, the Netherlands; 2 Faculty of Architecture / Delft University of Technology / PO Box 5043, 2600 GA Delft, the Netherlands; E-Mail: E.T.Schutte-Postma@tudelft.nl

**Keywords:** Particulate Matter, air quality, environmental policy, spatial planning, zoning, European Union

## Abstract

The distribution of Particulate Matter in the atmosphere, resulting from emissions produced by cars, trucks, ships, industrial estates and agricultural complexes, is a topical public health problem that has increased in recent decades due to environmental factors in advanced economies in particular. This contribution relates the health impact caused by concentrations of Particulate Matter (PM) in ambient air to the PM standards, the size of the particles and spatial planning. Diverging impacts of PM standards in legal regulation are discussed. The authors present a review of the development of legal PM standards in the European Union, with a specific reference to The Netherlands.

## 1. Introduction

In developed countries, many public health problems appear not only to have been identified but also largely solved. The quality of drinking water supplies and sewerage systems has made a particularly important contribution to improving public health. However, the prosperity of the developed world also gives rise to new public health problems, such as the increase in obesity in countries where children and adults eat too much, eat unhealthy products and exercise too little. A far-reaching dependency on the car as a means of transport is a key contributing factor.

Another topical public health problem that has received more attention in recent decades due to environmental factors in advanced economies is the distribution of Particulate Matter (PM) in the air, resulting from emissions produced by cars, trucks, ships, industrial estates and agricultural complexes. This contribution provides an overview of this crucial aspect of air quality, in which environment and public health come together, and deals with a number of research questions.

The first of these, the impact of high PM concentrations on public health, is dealt with in the remainder of this Section. This section illustrates the empirical evidence with regard to this topic.

Section 2 presents a general overview of European efforts to regulate air quality, with a special focus on PM concentrations. This section addresses the research question as to how regulation of PM_10_ concentrations in Europe has evolved since about 2000. After that especially the situation in the Netherlands is discussed: in particular the way the Netherlands transposed the EC regulation on PM.

The Netherlands initially sought to forge a strong relationship between PM standards and different aspects of spatial planning: infrastructure planning, urban planning and land-use planning. In the first few years, this approach led to many construction and infrastructure projects being halted for failing to meet the relevant PM standards. Section 3 examines the research question of how to evaluate PM standards in Europe and the Netherlands.

PM_10_ (‘Particulate Matter’) is a blanket term for all the tiny specks of dust with a size of 10 micrometres (10 microns) or less floating around in the air. These particles are so minute that they can become embedded in the lungs and damage health. Around 54% of Particulate Matter in the Netherlands consists of soil particles and ‘natural’ particles resulting from a coastal climate: sand and sea salt aerosols. The other 46% consists of all sorts of unnatural substances, such as polycyclic aromatic hydrocarbons (PAH). PM_10_ is produced by, amongst others, exhaust gases from motorised vehicles, domestic heating and by agriculture, ships and industry. These particles (soot!), emitted mainly by industry, traffic and domestic heating, can cause asthma, cardiovascular problems, lung cancer and premature death. A further distinction is made between coarse particles (2.5–10 microns), fine particles (less than 2.5 microns) and ultrafine particles. It is the tiniest particles that present the greatest risk for human health.

The air above the Randstad Holland and Southern Netherlands is among the dirtiest and unhealthiest in the whole of Europe (see Figure 2). Around one third (31%) of the polluted air comes from industrial belts across the border in Germany, Belgium and the UK. On the other hand, the Dutch export three times as much polluted air as they import and are certainly co-responsible for a major part of the cross-border air pollution.

On average, only 6% of all Particulate Matter in the Netherlands is caused by local traffic. The combined influence of industry, mobile machinery, consumers and agriculture is less than 10%. So, taken together, the Dutch sources account for an average of 15%. The percentage from (local) traffic is higher in the cities. The contribution from Dutch sources can rise locally to 45% [1]. In conclusion: the bulk of particulates in the Netherlands occur naturally (for a part benign) or are imported.

**Figure 1.**
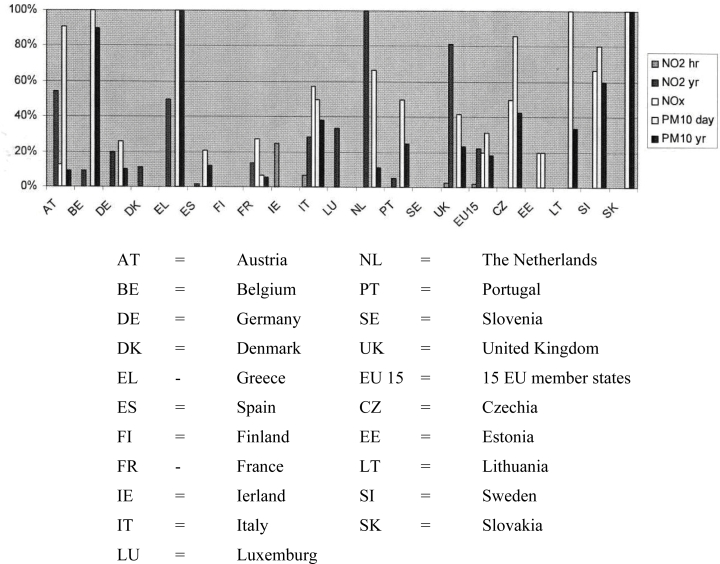
Percentage of zones exceeding the limit value (plus margin of tolerance if existing) for NO_2_/NO_x_ and PM_10_ in 2003 [2].

Under the European standards, effective from 1 January 2005, the maximum annual average for PM_10_ is 40 micrograms per cubic metre. The 24-hour average may not exceed 50 microns per cubic metre more than 35 times a year. Large parts of the Netherlands (including local sites) are unable to meet these standards (especially the 24-hour standards). The same applies for some other EU countries (Figure 1).

In the USA PM first came into the spotlight in 1974 as a result of the ‘Harvard Six Cities’ research project. This project led to a much talked-about article by Douglas Dockery and others in the *New England Journal of Medicine*, which showed that even low concentrations could be fatal [3]. It appears that PM, or the particles that accompany them, were to blame for the extra mortality observed in the US states. The study revealed a relationship between air pollution and deaths from lung cancer and cardiac and pulmonary disease.

Since 1974, there has been a flood of epidemiological reports on the effects of PM. Some are fairly simplistic, others are more refined and introduce corrections for smoking habits, diet and other ‘spurious factors’. In 2000 Jonathan Samet and others from Johns Hopkins University in Baltimore showed that out of the five potentially toxic substances in urban air (PM, ozone, CO, SO_2_ and NO_2_) PM alone were clearly to blame for extra mortality [4].

Research by Van Schayck, Professor of Preventive Medicine at Maastricht University (the Netherlands), revealed that the particles that form the most serious health risks for schoolchildren are polyaromatic hydrocarbons (PAH) and heavy metals released by incineration and combustion processes in industry and traffic [5]. These particles damage the cell membranes in the lungs and cause infection.

His research also revealed that the pulmonary function of 342 children was 15% below the predicted level. No difference was found, however, between the pupils from two schools along the busy A2 motorway that crosses Maastricht and the pupils from a school on the quiet west side of the city. This may be because Maastricht as a whole is covered by a blanket of fine particles that hangs over the Maas valley [5].

It was clear that exposure to PM with a “high capacity for generating radicals” was having a demonstrable effect on the pulmonary function of the 131 children who had lived their whole life in the direct vicinity of their school and were constantly exposed to the highly aggressive PM. The correlation between pulmonary function and the smallest toxic PM was six times higher for them than for other children. The smallest bronchi were especially affected. It is the extremely fine PM that causes the most damage, as they can penetrate deep into the tiny threads of the bronchioli.

According to Van Schayck *et al.* [5], the EU directive was far too general before 2006. It should not concern itself with PM_10_ but with PM_2.5_: particles with a diameter of 2.5 micrometres and less. These are included in the European PM standards recently. Finally, the Maastricht research underscores the precariousness of trying to establish a direct connection between air quality and specific locations.

Nevertheless, Dutch environmentalists argue that the rigid Particulate Matter standards need to be maintained in the interests of public health. Though the sceptics argue otherwise [6,7], ignoring the Particulate Matter standards could cost lives [8,9]. According to the Netherlands Environmental Assessment Agency (*Milieu en Natuur Planbureau MNP*), between 2300 and 3500 people die prematurely in the Netherlands each year as a result of short-term exposure to particulates. The effects of long-term exposure are much more serious with estimated mortality rates in the region of 12,000 – 24,000. Casualties resulting from exposure to PM may be just as high as casualties from road traffic accidents, obesity and passive smoking [1,10–12]. In comparison: the EC Thematic Strategy on air pollution [COM(2005) 446 final] says: “There is currently in the EU a loss in statistical life expectancy of over 8 months due to PM2.5 in air, equivalent to 3.6 million years of life lost annually. Even with effective implementation of current policies this will fall only to around 5.5 months (equivalent to 2.5 million years of life lost or 272,000 premature deaths)”.

The estimates of the Netherlands Environmental Assessment Agency are based on epidemiological research which looks for statistical correlations between high concentrations of airborne particulates and premature death and/or reduced pulmonary capacity. However, Crok wrote an article intriguingly entitled ‘Where are the deaths from particulates?’ [6]. His answer is: nowhere. So far, Particulate Matter has not been formally identified as a cause of death in individual cases. This reasoning is blatantly wrong because even for very evident cases such as the big London fog [13], a doctor can not diagnose, which cardiovascular disease was caused by air pollution and which by other reasons, even if an epidemiologist can provide the information that e.g. 70% of the cases are caused by air pollution. This is related to the toxicological mechanism of PM, which does not leave a ‘fingerprint’. Instead, PM increases inflammation and acute phase substances, which subsequently lead to an adverse event [14].

A study by Singels *et al*. [11] quantifies the public costs of damage to health in the Netherlands. The health effects (death and illness) of short-term exposure to particulates and ozone cost 100–400 million euros a year in the Netherlands alone. The overall health effects of long-term exposure to particulates cost at least 4 billion euros a year and could soar to an astronomical 40 billion. The figures for short-term exposure are fairly sound, those for long-term exposure are open to question.

## 2. European PM_10_ Monitoring and Regulation

Below the regional PM_10_ concentrations in Europe will be shown in order to give an impression of the situation which is ‘standard related’. Subsequently the European air quality regulation on PM_10_ concentrations and standards are discussed together with their legal backgrounds and the different interpretations of that regulation by the member states.

### 2.1. Levels of PM_10_ Emissions in Europe

**Figure 2.**
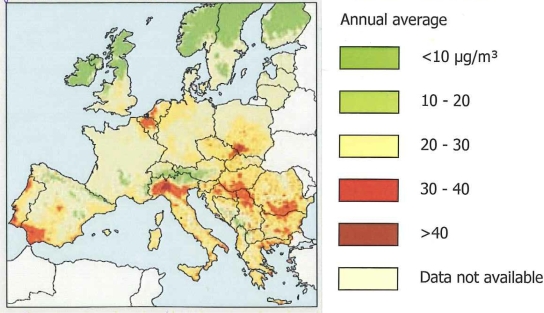
Regional PM_10_ concentrations in Europe, 2004. Source: (Milieubalans 2007, Milieu- en Natuurplanbureau (MNP); Publ. Nr. 50081004, Netherlands [15].

Figure 2 presents the regional PM_10_ concentrations in Europe as at 2002. We observe high concentrations in economic centres and urban regions such as the Po Delta in Italy, Paris and the surroundings and, above all, the Randstad (the Netherlands), the Flemish Diamond and the Rhein-Ruhr area. Note that from Paris and big parts of Germany no data were available.

**Figure 3.**
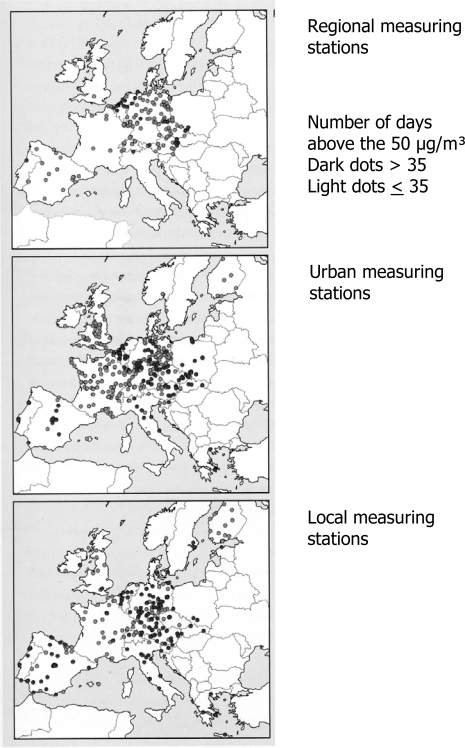
Overruns of limit value for daily average PM_10_ concentration in Europe, 2002. [About here: Figure 3. Overruns of limit value for average daily PM_10_ concentrations in Europe, 2002] Source: MNP, 2005 [1].

Figure 3 shows the overruns of the limit value for average daily PM_10_ concentrations in Europe, 2002. Overruns seem to be more the rule than the exception.

**Figure 4.**
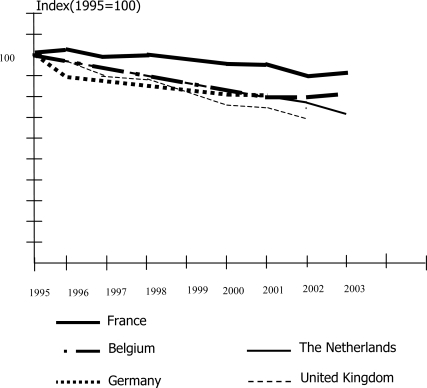
Emissions PM_10_, 1995–2003. [About here: Figure 4. Emissions PM_10_, 1995–2003] Source: MNP, 2005 [1].

Figure 4 gives an overview of the emissions of PM_10_ in 1995–2003 in five European countries. There is a clear trend of gradually decreasing concentrations of PM_10_.

**Figure 5.**
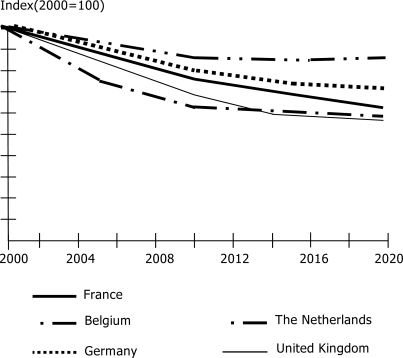
Emissions PM_10_, 2000–2020. [About here: Figure 5. Emissions PM_10_, 2000–2020] Source: MNP, 2005 [1].

This trend of decreasing concentrations is expected to continue in the next decades, as shown in Figure 5.

Given that the real emissions of PM_10_ exceed the standards for public health, it is no surprise that the European Commission has accepted responsibility for the regulation of air quality, including the regulation of PM_10_ concentrations.

### 2.2. European Air Quality Regulation on PM_10_ Concentrations

The legal framework for air quality standards consists of the Directive on Ambient Air Quality and Cleaner Air for Europe of the European Parliament and of the Council of 21 May 2008 (2008/50/EC) which came into force 21 June 2008. This directive is the successor - and in fact a combination - of several former directives which were basic for the regulation of PM_10_ concentrations in the foregoing period since 2001. For a good understanding these foregoing directives will shortly be discussed below together with the present Directive (2008/50/EC) and a few important changes which have been made in it.

### 2.3. Legal Background of the Particulate Matter Standards, the ‘Framework Directive’

As stated before the legal framework for air quality standards consists of the Directive on Ambient Air Quality and Cleaner Air for Europe (2008/50/EC) which as such forms an integration of former directives. These are the *Directive on Ambient Air Quality Assessment and Management* (96/62/EC), known as the *Air Quality Framework Directive* and four ‘daughter directives’ which set out the concrete implications (standards) and requirements for 13 substances as laid down in the Framework Directive. The Framework Directive ordered the Commission to formulate new concept directives for 13 substances and PM_10_ was one of those. The standards for PM_10_ have been set out in the so called ‘*first daughter directive’* (1999/30/EC), the Council Directive 1999/30/EC of 22 April 1999 relating to limit values for sulphur dioxide, nitrogen dioxide and oxides of nitrogen, particulate matter and lead in ambient air. All mentioned standards become part of the new ‘directive on ambient air quality and cleaner air for Europe’ now. The standards are based on WHO health research.

The Framework Directive (96/62/EC) required among other things the member states to draw up action plans or programmes for attaining the standards (limit values for PM_10_). Chapter IV ‘Plans’ of the ‘new directive’ picks this up in ‘Air quality plans’ (Art. 23) and ‘Short-term action plans’ (Art. 24). In case of exceedance of a limit value for which the attainment deadline is already expired the air quality plan shall set out appropriate measures, so that the exceedance period can be kept as short as possible. Air quality plans may additionally include specific measures aiming at the protection of sensitive population groups, including children.

### 2.4. The ‘First Daughter Directive’ (1999/30/EC)

The first daughter directive dates from 1999 and setted ceilings (limit values) for five substances including PM_10_. It also made reference to PM_2.5_ and instructed the member states to commission research into these substances. The implementation deadline was 19 July 2001. There were no standards for PM_2.5_ – a strange state of affairs considering that there has been much concern about PM_2.5_ standards in California since the 1980s. European standards for PM_2.5_ were finally set out in the new Directive on Ambient Air Quality and Cleaner Air for Europe (see section 2.7 below).

#### Important points concerning PM_10_:

1. *Air quality standards for Particulate Matter*. Member states must take the measures necessary to ensure that the concentrations of PM_10_ do not exceed the limit values (1999/30/EC Art. 5; 2008/50/EC: Art. 13, chapter III *Ambient air quality management*). The first limit value, a 24–hour value to protect human health, is set at 50 micrograms of PM_10_ per m^3^ and may not be exceeded more than 35 times a year. The second, an annual value to protect human health, is set at 40 micrograms per m^3^. Both limit values had to be attained by 1 January 2005.These standards are still the same and set out in Annex XI to the new directive.
2. *Exceptions to limit values* are possible if *natural sources* cause the concentrations to significantly exceed normal background levels. In such cases the member state must inform the Commission and submit evidence. The member state may be relieved of its obligation to take action if the pollution is caused by *the resuspension of PM* (‘following the winter sanding of roads’) provided it informs the Commission of the situation and justifies its claims (1999/30/EC: Art. 5.4 and 5.5). Sweden has been successfully using this exemption clause since 2001. The Netherlands did not pick up on it until 2005. The new directive sets out a comparable regulation (2008/50/EC Art. 20 and 21).
3. *Division into ‘zones and agglomerations.’* The member states had to divide their territory into measurement zones and agglomerations which matched specific criteria in the directive. EC member states differ in their allocation policy: some defined only a few, very large zones, others allocated much more differentiated zones over their territory.
  Agglomerations are urban areas with a population of over 250,000, or a density per km^2^ which warrants air quality assessment and management by the member states. Member states review the demarcation lines of zones and agglomerations once every five years or earlier, depending on scientific developments (1999/30/EC: Article 7.1). Directive 2008/50/EC is similar on this point (Art. 5 and 6 Assessment regime resp. Assessment criteria).
4. *Assessment* and *measurement.* The directive determines the *location* of the sampling points (in general 4 metres from the motorway) and the measurement *methods* (1999/30/EC: Article 7 and Annexes VI and VII). It also stipulates the situations in which *measurements* are to be taken and those in which certain *calculation methods* are deemed adequate. Sometimes, a combination of measurement and calculation is allowed. Annex III and Annex VI to Directive 2008/50/EC seem to be comparable.
5. *Public information.* The general public and ‘appropriate’ organisations must always have access to up-to-date information on air quality (1999/30/EC: Article 8; 2008/50/EC: Art. 26). ‘Appropriate’ organisations are, for example, environmental and consumer organisations and interest groups for patients with chronical aspecific respiratoric diseases. This information must be updated every day. Information on NO_2_ and PM_10_ is posted on national websites all over the EC territory.
6. *Reporting.* The member states are required to report their experience to the Commission (1999/30/EC: Article 10; 2008/50/EC: Art. 27).

Any member state that fails to meet the limit values is obliged to prepare a *plan* or *programme* to attain them (Article 8, Framework Directive 96/62/EC; 2008/50/EC: Art. 23 and 24).

It may be clear that the directive on Ambient Air Quality and Cleaner Air for Europe (2008/50/EC) is similar to both mentioned former directives to a large extent. There are however two main differences: one related to a standard for PM_2.5_ and the other to a possibility of postponement of attainment deadlines for the obligation to apply certain limit values. These will be discussed later.

### 2.5. Air-Quality Standards: Limit Values and Target Values

There are different kinds of legal air quality standards with different implications. A distinction is drawn between ‘limit values’ and ‘target values’. Limit values legally imply a ‘results obligation’ as they must actually be attained by the member states. Target values imply an ‘effort obligation’ in so far as the member states need to make (only) substantial ‘efforts’ to attain them. Hence, limit values are more stringent than target values.

Most of the standards in air quality directive 2008/50/EC (except for ozone; for PM_2.5_ see below) are limit values, based on the protection of human health and the environment (Article 175, EC Treaty). The health standards are derived from the Air Quality Guidelines of the World Health Organisation. The importance of health is reflected in the objectives of the European environmental policy as laid down in Chapter XIX (Environment, Article 174.1) of the EC Treaty. The standards in air quality directive 2008/50/EC are minimum standards. The member states are at liberty to tighten them for children and other vulnerable groups. Recently in the Netherlands an administrative order came into force (16 January 2009) to prevent the building of schools and various other projects for vulnerable groups like children and elderly people near motorways (< 300 meters) or provincial highways (< 50 meters) [16].

### 2.6. Measurement and Calculation

Air quality measurement and calculation form an important part of the directives. There are two main methods for ascertaining and assessing air quality: measurement and model calculation, often used in combination. Strictly speaking, measurements should be recorded across the entire territory of the member state. When concentrations are close to the limit values, models may be used in combination with measurements. The criteria for the measurements are set out in the first daughter directive (Annexes VI and VII). Professional opinions are divided on the methods used [17,18]. A discussion is underway concerning the variety of methods and models used by the member states. It is almost impossible to objectively compare the results of extrapolations for the future, which cannot be verified by measured data.

Model calculations are mathematical computer simulations of the dispersion of substances in the atmosphere. The core question with regard to measurements is whether the right sampling points have been selected. The core question in model calculations is validity. The legal criteria are generous (margins of 50% are allowed) as there was scarcely any experience of the use of models in this area.

The accuracy of the measurements and the calculations depends largely on the expertise of the people concerned. Van den Hout makes the general observation that experience has shown that errors are not uncommon even when standard techniques are applied [17]. There are indeed many different measuring and modelling techniques, some of dubious accuracy and validity. One crucial factor is the prognostic analysis which estimates the effects of the intended measures. Because the techniques are fraught with uncertainties, they cannot always support the conclusions drawn.

To determine future air quality (prognostic analysis) it is necessary to work out the overall effects of new developments on pollution (e.g. growth of traffic) and the effect of any planned measures to reduce emissions. The daughter directive offers no guidelines (or calculation models) in this area, leaving the member states free to make their own decisions. Obviously, it is in their interests to use well-tried methods. Over-optimistic calculations carry the risk of sanctions if the limit values are not achieved, while over-pessimistic estimations could prove costly [17]. As might be expected, with no guidelines and with freedom of choice, the prognostic analysis methods in the EU are fairly diverse. (See for a prognostic study with two scenarios, 2000 – 2030 for 20 European cities [19]).

### 2.7. New Aspects in the Directive on ‘Ambient Air Quality and Cleaner Air for Europe’

#### Standard for PM_2.5_

A standard for PM_2.5_ has finally been adopted in this directive. This standard is defined as an *exposure reduction target and concentration cap* especially in *urban background locations* (a 15 or 20% reduction to be attained by 2020 with 2010 as the reference year). What this amounts to in legal terms is a special kind of ‘*effort obligation’*, which is less rigorous than a results obligation (limit value). A *target value* like this seems a good starting point for establishing a new standard. There is also a *general limit value* (25 micrograms of PM_2.5_ per m^3^ in 2015) which will start off as a target value in 2010.

#### Postponement of deadline to apply certain limit values

New is the legal possibility of postponement of the deadlines if conformity with the limit values cannot be achieved in time. This is also possible for the deadlines for PM_10_ which had to be achieved at 1 January 2005 already. Where in a given zone or agglomeration conformity with the limit values for PM_10_ cannot be achieved because of site-specific dispersion characteristics, adverse climate conditions or transboundary contributions a Member State shall be exempt from the obligation to apply those limit values until 11 June 2011. Member States however should have established an air quality plan for the area and should show that all appropriate measures have been taken at national, regional and local level to meet the deadlines. This is a practical solution for the fact that almost all Member States (except for Luxembourg and Ireland) did not meet the PM_10_ standards in time. So far the Commission started infringement proceedings against 10 Member States to comply with the standard for PM_10_ [20]. Eleven Member States have so far notified requests for time extensions. For people’s health however the postponement provision could turn out worse of course.

#### Air quality and spatial planning

One striking aspect of the European Air Quality regulation is that it makes no connection between air quality guidelines and spatial planning. On the other hand, it does not specifically deny the existence of any such connection. In the EC Treaty the term ‘environment’ tends to be very broadly interpreted and might also include spatial planning. In general ‘good spatial planning’ requires a spatial assessment of zones on the basis of research – also into air quality – in the area that is due to be developed. Hence, it safeguards against designations which are irresponsible in terms of air quality. The Netherlands is the only country in the European Union to have *legally* linked air quality standards and spatial planning. The most rigid link was laid down in the 2001–2005 legislation. This explicit legal link meant, however, that from mid 2004–2007 almost every spatial decision had to be tested against the air quality standards, so also against the standards for PM_10_ (in force since 1 January 2005). Measures were needed to ensure that they were met: if this turned out to be impossible the administrative judge annulled the plan. First in August 2005 and later in 2007 Dutch legislation has been changed into a more ‘nuanced’ regulation which gave some more flexibility.

#### Ambiguity surrounding interpretations of the European regulations

Implementation of the air quality standards by the member states led to a wide array of legislation. In some countries (e.g. Austria) implementation was more rigorous than required by the air quality directives. The UK and Sweden introduced the standards before the deadline and France applied the exceedance margins in the directive (NO_2_) as limit values while other member states were applying them as target values.

Moreover, the obligation to ascertain air quality is interpreted in many different ways by the member states. As a result, there are wide discrepancies in the pictures derived from the measurements: model calculations for concentrations at street level (Denmark, UK, Sweden; see [19]), or measurements or models that do not calculate concentrations at street level (Germany, Greece, Portugal). Spain even gets by with calculations for SO_2_.

Interpretation of European legislation is a task for the Commission, the European Court of Justice, and the legislators and courts in the member states. European environmental legislation sometimes gave rise to conflicts and uncertainty about interpretation. For example, the interpretation of the Habitats Directive 92/43/EC on the conservation of natural habitats and wild fauna and flora (nature preservation, Natura 2000) and the Environmental Impact Assessment Directive 85/337/EC (obligation to assess environmental impacts of potential harmful projects) created much jurisprudence on spatial planning from the European Court of Justice (ECJ) and the national courts as well. Air Quality Directives also caused uncertainty. These directives appeared to have lost a lot in translation when they were integrated into the national legislation of the member states. Moreover, there was no consistency in the role of the national court in the member states. In Germany, France, Belgium, Austria and the UK only a few cases have been referred for judgment compared with many in the Netherlands. So, there are quantitative and ‘qualitative’ aspects. National courts in the Netherlands have stringently tested many infrastructure projects and other spatial plans against Dutch air quality legislation even before 1 January 2005 (in force date PM_10_ standard), often with negative outcomes [21–23] (e.g. ABRvS 22.09.2004 Hendrik Ido Ambacht, 200307780; ABRvS 09.02.2005 Zoningplan Stationseiland Amsterdam, 200400323/1; ABRvS 22.11.2004 Zoningplan Gershwin Amsterdam, 200406190/2; ABRvS 05.04.2006 FOC Roosendaal, 200506157/1). Other (national) jurisprudence seems to be more flexible (e.g. Germany [21]). In contrast, the role of the European Court of Justice seems to have been fairly modest so far (see below).

At least in the Netherlands implementation of the air quality policy is accompanied by an extraordinary degree of legal ambiguity. In the Netherlands and Germany, for instance, expert opinions differ on what is and is not allowed under European Law.

European Law is supreme. It must be administered by all national governments and courts (Article 10, EC Treaty). Any member state that fails to attain the limit values (in force) is in breach of the law. The standards are stringent and uniform. Member states were required already under the Framework Directive to draw up and implement action plans for the eventual attainment of any standards which are not (or are unlikely to be) met. The new directive on ‘Ambient Air Quality and Cleaner Air for Europe’ (Air Quality Directive 2008) holds the possibility of postponement in the attainment of limit values (see above), an option which the Netherlands is trying to use already. A request of the Dutch Government together with a national air quality plan (Nationaal Samenwerkingsprogramma Luchtkwaliteit; National Cooperation Program on Air Quality; government stand) has been presented July 2008 to the European Commission. April 2009 it is expected to be clear if the Commission will agree to the request.

Any legal conflicts on the attainment of standards are submitted to the court – usually the national (administrative) court or – in some cases – the European Court of Justice in Luxembourg. The Commission also plays a role in the interpretation of the directives and the standards. Further execution of the directives takes place largely upon the initiative of the Commission. It is also the Commission’s task to make the member states implement and apply the directives. To fulfil this task the Commission might bring a case against a member state at the European Court of Justice. In the event of a conflict (between the Commission and a member state on the interpretation of EC law), it is the European Court that decides. If necessary, the European Court can correct the Commission if it decides that the Commission has wrongly interpreted a directive. This is only possible if the Court is asked to make a judgment. This could be requested by the Commission in the event of a dispute with a member state (or between member states). Also, a national court may put a so-called ‘preliminary question’ to the European Court of Justice if it is unsure about the interpretation of European law in the judgment of a case. However, actions like this tend to take a very long time.

Until July 2008, the European Court of Justice has made only one important judgment on the interpretation of Particulate Matter standards (Case C-320/03, Commission v. Austria; November 2005). The case in question concerned a decree in Tyrol (Austria), which banned heavy traffic (750 tons and over) from a stretch of the Inntal Autobahn because it was allegedly affecting the air quality. The Commission, Germany and Italy had complained about the ban. The European Court overruled the decree primarily because the province had failed to adequately explore alternative ways of preventing the pollution before resorting to such drastic measures. The Court also took the view that the action was not part of a wider strategic plan to improve the air quality. What is more, haulage firms were only given three months to solve the problem. The Court regarded the entire exercise as careless. The background issue was that, according to the Commission, Germany and Italy, one of the European freedoms was being infringed, *viz*. the freedom to transport goods. It may however be inferred from the Court’s decision that it is, *under certain conditions, perfectly acceptable to take drastic steps to attain air quality norms projecting human health – even if they encroach on the right to transport goods*. It is also worth noting that the European Court of Justice demands a cohesive strategic plan as stated in the Framework Directive.

One reference for a ‘preliminary question’ was submitted to the Court (by the German *Bundesverwaltungsgericht*) regarding the interpretation of the action plans (in the Framework Directive) required from public authorities to improve air quality in relation to the health of third parties (Case C-237/07, Janecek v. Freistaat Bayern). The Court’s judgment (25 July 2008) was that where there is a risk that limit values may be exceeded persons directly concerned can require the authorities to draw up an action plan .The authorities have some discretion in deciding the measures and time schedule in the plan (gradual return to a level below the values, taking into account the factual circumstances and opposing interests).

#### Quality of environmental legislation

A debate is currently underway on the quality of European environmental legislation. At European level the quality of the standard-setting should be checked and maintained more with models and monitoring. The current air-quality regulations are difficult to enforce because the transposed national regulations of the member states, regions and local authorities have become extremely complex and opaque [24]. The EU regulations provide good guidelines for member states who want to reduce emissions. It would be very difficult – perhaps even impossible – to enforce the EU legislation on member states who attach less importance to air quality. On the other hand, there is a body of opinion which states that a different, more flexible ‘bottom-up’ procedure would be needed to realise the environmental policy of the European Union – now comprising 27 member states.

## 3. Some Notes on European and Dutch Particulate Matter Standards

It would be no exaggeration to say that, for several years, Particulate Matter standards have caused mayhem among spatial developers, infrastructure planners, builders and local authorities in the Netherlands. The obvious conclusion is that, initially, the link between the European directive on Particulate Matter standards and Dutch national spatial plans and projects was far too rigid. The Netherlands is the only country in the European Union where such a rigid legal connection between Particulate Matter standards and spatial development could be observed. The new Dutch air quality legislation has been changed radically in two steps: a new Air quality Decree 2005 (August 2005) later replaced by a new Air Quality Bill (a wish of Parliament) in December 2007. This legislation is more flexibly linked to a National Programme for Collaboration on Air Quality, due to come into force in 2009. Under this 2007 legislation only in exceptional and critical situations will extra measures be needed to prevent industrial complexes, infrastructure projects (both as producers of emissions) and conventional buildings (as attractors of traffic) from exceeding the air quality standards.

Dutch spatial planning legislation also empowers local authorities to use their zoning plans to prevent designations which would be irresponsible in terms of air quality (no vulnerable designations at polluted locations). At present Dutch air quality legislation only regulates the limitation of emissions from activities.

In the discussions about PM standards politicians sometimes make a distinction between benign and toxis particles. However, this distinction has several basic flaws. It is well established in toxicology that the effects of the substance should be considered. Indeed, some of the most toxic substances are of “natural” origin (e.g. botulinus, aflatozin and curare). Furthermore, even seemingly harmless particles such as salt spray can act as a carrier for other toxic air pollutants and salts can provide the substrate to form highly aggressive secondary pollutants. These issues have been extendedly been discussed before the introduction of the new directive, also within the scientific community: COST Action 633 concluded that source-related heterogeneities can be observed for cardiovascular or respiratory mortality and morbidity [25]. On the one hand there is currently not enough scientific evidence to declare any source or chemical composition as “non-toxic”, on the other hand there are large differences in toxicity of different components in a heterogeneous mix of Particulate Matter [26].

In large parts of West and South Netherlands the Particulate Matter concentrations (expressed in PM_10_, regardless of being toxic or not) were recently above the European limit values for the daily average. Until recently this seemed to result in a totally pointless ban that could last for years. In *Eigen Huis* [27] State Secretary Pieter Van Geel even suggested that building would have to take place temporarily elsewhere in the Netherlands. He later re-phrased this comment in more diplomatic terms. Since then Dutch government has gone to great lengths to avoid such a catastrophic fate (“The Netherlands is off-limits”).

It is remarkable that the implementation of air quality standards was nowhere near as draconian in any EU member state as it was in the Netherlands. This may (partly) be explained by the wider legal protection in the Netherlands, based on the General Administrative Law Act (*Algemene wet bestuursrecht*) [21]. Apparently, most of the other member states were able to adopt a more pragmatic approach. The steps taken by countries such as Germany and the UK to discourage traffic from entering city centres are partly aimed at improving air quality (nine big cities in Germany; the City of London). Obviously, these do not need to be tested against spatial criteria.

One could conclude that, initially, the Dutch authorities failed to convert the European regulations on Particulate Matter into national legislation in an intelligent manner although possibly aiming at better results to health. Given the relatively strong concentrations of air pollution in this part of Europe, the Dutch government should have foreseen that application of the limit values for PM_10_ would lead to many exceedances. It is therefore difficult to fathom why the Dutch government did not, at the very start, suggest corrections for Particulate Matter with a less toxic, natural background such as sea salt, as the Swedish government did. To complicate matters further, the Dutch government initially assumed that virtually every building plan would have to be tested against the PM_10_ standards. On this point the Netherlands isolated itself from the rest of Europe. If, in large parts of the country, the PM_10_ standards were already being exceeded *without* a building plan, one could safely assume that they would be exceeded *with* a building plan. The Court had no choice but to apply the Dutch Law and therefore stopped in 2005 dozens of building plans at locations where the limit value was exceeded and where the initiators had done only perfunctory research or none at all.

From a number of evaluations it has further emerged that, apart from a small circle of environmental officials, hardly anyone in the Netherlands realised that new air quality standards had been introduced [28,29]. No information or training programmes were organised. Is it any wonder then that almost all the professionals in the sector were dumbfounded when the first building projects were stopped by the Council of State?

Later the Dutch government opted for a - from an urban building planning point of view - more pragmatic approach. All those involved could now contribute to finding creative solutions to the problems. Henceforth, only the larger building plans would be tested against the new standards. Under the banner of ‘better late than never’ the Dutch government took advantage of the correction possibilities for natural particles which the European regulations had offered from the start. And the introduction of a trading system allowed it to be more supplied in the application of the limit values. By easing the link between Particulate Matter norms and spatial policy the Dutch government was able to create a more workable situation. The isolated situation that it had initially chosen did not go down well with Dutch builders and planners.

Dutch policy in this domain may be severely criticised. A few critical comments can also be made regarding the principles of the European policy on Particulate Matter, which definitely improved in December 2007 with the advent of the new Air Quality Directive and the standards for PM_2.5_. We have singled out three points of criticism:

1. The European standards for maximum concentrations of Particulate Matter make no distinction between more and less toxic particulates. In a later stage differences in concentrations of sea salt are (more or less) taken into account in the Netherlands. We argue that a more valid approach should be followed: the standards should relate only to toxic particulates. This is not a matter of black and white. The share of toxic particulates can fluctuate strongly depending on the location. When an approach would be adopted, in which the difference between toxic and less toxic (mostly “natural”, such as sea salt) particles would be taken into account, then the risk calculations would need to be repeated on the basis of dose-response curves that follow the same concept, i.e. PM-measurements for which the natural background was deducted. This could very evidently result in steeper dose-effect curves and thus in lower PM-standards. It is likely that the resulting non-attainment numbers would be very similar. However, before any such procedure can be conducted, all the scientific information justifying and underpinning this approach would need to be created. This means: more research would be necessary.
2. There is considerable legal tension between the current precisely defined PM_10_ and PM_2.5_ standards and the obscurity surrounding measurement and modelling techniques and the health effects of the different fractions in the Particulate Matter. Given the current status of research, the strict PM_10_ standards expressed in the limit values are too rigid. More flexibility should be needed, and the different interests must be weighed more evenly. Environmental standards in absolute figures may stretch precaution too far. The European Parliament was also aware of these effects when discussing the new directive on ‘Ambient Air Quality and Cleaner Air for Europe’. This is certainly true as long as the EC source policy (traffic, fuel etc.) is not in line with a strict ambient air quality policy.
3. The legal uncertainty caused by ambiguous regulations and the lack of clear jurisprudence from the European Court of Justice had a negative impact on spatial development in member states which intended to rigorously implement the regulation.

So far air quality policy of the European Union is formulated in limit values for PM_10_ and in certain areas target values for PM_2.5_. This relates air quality policy primarily to spatial policy and only indirectly to health. Since 2004 the power of spatial planning to deal with PM has been overestimated in the Netherlands. Later on the relations between spatial planning and air quality standards have been made more flexible. Public health in the EU member states could be particularly well-served when there is an ambitious, direct trans-border *source policy* which, in terms of effectiveness, will overshadow all the present measures. The European Parliament has played a crucial role in the political debate on this topic. Hopefully, source policy will be at the heart of Particulate Matter standards and air quality policies in the future.
